# Uranium Biogeochemistry in the Rhizosphere of a Contaminated Wetland

**DOI:** 10.1021/acs.est.3c10481

**Published:** 2024-03-28

**Authors:** Daniel I. Kaplan, Maxim I. Boyanov, Nathaniel A. Losey, Peng Lin, Chen Xu, Edward J. O’Loughlin, Peter H. Santschi, Wei Xing, Wendy W. Kuhne, Kenneth M. Kemner

**Affiliations:** † Savannah River Ecology Laboratory, University of Georgia, Aiken, South Carolina 29802, United States; ‡ Argonne National Laboratory, Lemont, Illinois 60439, United States; § Chemical Engineering, Bulgarian Academy of Sciences, Sofia 1040, Bulgaria; ∥ Savannah River National Laboratory, Aiken, South Carolina 29808, United States; ⊥ Marine & Coastal Environmental Science, Texas A&M University − Galveston, Galveston, Texas 77553, United States

**Keywords:** XAFS, roots, oxidation state, 16S rRNA, speciation

## Abstract

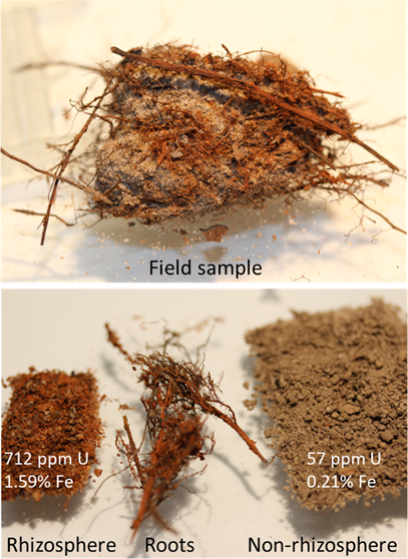

The objective of this study was to determine if U sediment concentrations in a U-contaminated wetland located within the Savannah River Site, South Carolina, were greater in the rhizosphere than in the nonrhizosphere. U concentrations were as much as 1100% greater in the rhizosphere than in the nonrhizosphere fractions; however and importantly, not all paired samples followed this trend. Iron (but not C, N, or S) concentrations were significantly enriched in the rhizosphere. XAS analyses showed that in both sediment fractions, U existed as UO_2_^2+^ coordinated with iron(III)-oxides and organic matter. A key difference between the two sediment fractions was that a larger proportion of U was adsorbed to Fe(III)-oxides, not organic matter, in the rhizosphere, where significantly greater total Fe concentrations and greater proportions of ferrihydrite and goethite existed. Based on 16S rRNA analyses, most bacterial sequences in both paired samples were heterotrophs, and population differences were consistent with the generally more oxidizing conditions in the rhizosphere. Finally, U was very strongly bound to the whole (unfractionated) sediments, with an average desorption *K*_d_ value (U_sediment_/U_aqueous_) of 3972 ± 1370 (mg-U/kg)/(mg-U/L). Together, these results indicate that the rhizosphere can greatly enrich U especially in wetland areas, where roots promote the formation of reactive Fe(III)-oxides.

## Abstract 2

Roots can alter the chemistry and mineralogy of wetland soils in a manner that increases the tendency for contaminant metals to become immobilized. These results have implications on the long-term stewardship of contaminated wetlands.

## Introduction

Wetlands have unique hydrological and biogeochemical properties that interact in a manner that makes them especially well-suited for sequestering organic and inorganic contaminants.^1−5^ Their unique hydric and fluctuating moisture conditions often support an especially wide diversity of plants. Furthermore, wetland sediments commonly act as net C sinks in the C cycle.^6^ The resulting elevated sediment organic matter (OM) concentrations provide several direct and indirect functions that enhance the capacity of wetland sediments to bind contaminants, including increasing microbial activity, acting as an electron donor, and creating more contaminant bonding sites.^7−9^

Plants alter the sediments they grow in and that sediment region is called the rhizosphere. The roots’ influence on sediment moisture reaches out for centimeters outside the root’s surface,^10^ whereas their influence on chemistry, mineralogy, and microbiology extends out only for a few millimeters.^11,12^ Many wetland plants have an adaptation to anoxic habitats due to their developed aerenchyma for the transportation of oxygen to roots and the surrounding sediment environment.^13,14^ The oxygen released by roots promotes, especially in wetlands, the oxidation of dissolved Fe(II) into a mixture of amorphous or crystalline Fe oxyhydroxides, mainly composed of goethite (α-FeOOH) and lepidocrocite (γ-FeOOH).^12,15−18^ The concentration of C can also be elevated in the rhizosphere from CO_2_, mucilage, exudates, sloughed-off root cells, or root dieback.^19−21^ Bacteria and fungi also release C into the rhizosphere, including siderophores, which are especially effective at chelating metals.^22,23^ When compared to the bulk sediment, the OM in the rhizosphere tends to be composed of molecules with greater overall molecular weights, less aromaticity, more carboxylate and N-containing COO functional groups, and a greater hydrophilic character.^24^

The greater dissolved oxygen, OM, and Fe-oxides content in the rhizosphere also influences the microbial populations, which have been studied extensively in agricultural and forestry settings^25−27^ and to a lesser extent in wetlands.^28−30^ A myriad of wetland-specialized rhizosphere microorganisms has been identified, including viruses, bacteria, archaea (such as N-fixers, nitrifiers, and methanotrophs), and fungi (such as mycorrhizal fungi), which together contribute to the ecological functioning of the wetland.^28^

The enrichment of Fe-oxides and OM in the rhizosphere provides a reactive surface for metal, metalloid, and radionuclide sequestration.^16,31−35^ Metals and metalloids found to be associated with iron root plaques include Mn, Zn, Pb, Cu, and As. Mn and Zn have been reported to exist as discrete, mixed-metal carbonate (rhodochrosite/hydrozincite) nodules on the root surface.^31^ In contrast, Pb has been reported to exist as organic complexes on iron root plaques.^31^

In previous studies, we demonstrated enrichment of U in the rhizosphere of two wetland plants, *Typha latifolia* (common cattail) and *Scirpus acutus*, grown under hydroponic conditions in which dissolved UO_2_^2+^ was introduced into a sand substrate maintained under reducing conditions.^35,36^ The objective of this study was to determine whether U was enriched within the rhizosphere under natural field conditions and to compare the speciation of U and Fe in the rhizosphere relative to the nonrhizosphere. Our hypothesis was that the ferric oxyhydroxides enriched in the rhizosphere of wetland plants are especially effective at binding metals. Our approach included collecting both U-contaminated and uncontaminated sediments from Tims Branch, a second-order stream located on the Savannah River Site in Aiken, South Carolina, that had received metal contaminants from a Nuclear Fuel Fabrication Facility between the 1950s and 1980s. The sediment samples were fractionated based on the color and proximity to roots into rhizosphere and nonrhizosphere paired fractions. These fractions were then characterized for total U, Fe, and Mn content by inductively coupled plasma-mass spectrometry (ICP-MS), organic C, organic N, and total S by combustion/inferred detection, microbial populations by 16S rRNA sequencing, Fe and U speciation by X-ray absorption fine structure (XAFS) analyses, and U sorption coefficients *(K*_d-desorb_ values, U_sediment_/U_aqueous_) by a batch desorption protocol.

## Materials and Methods

As noted above, portions of the Tims Branch study site are contaminated with U, and as such, radiological safety precautions were taken throughout the field campaign, laboratory handling of samples, analytical characterization, and shipping of these samples. Some experimental limitations associated with working with these radiological samples are identified when they impact the interpretation of the data.

### Field Sampling

**Figure 1:**
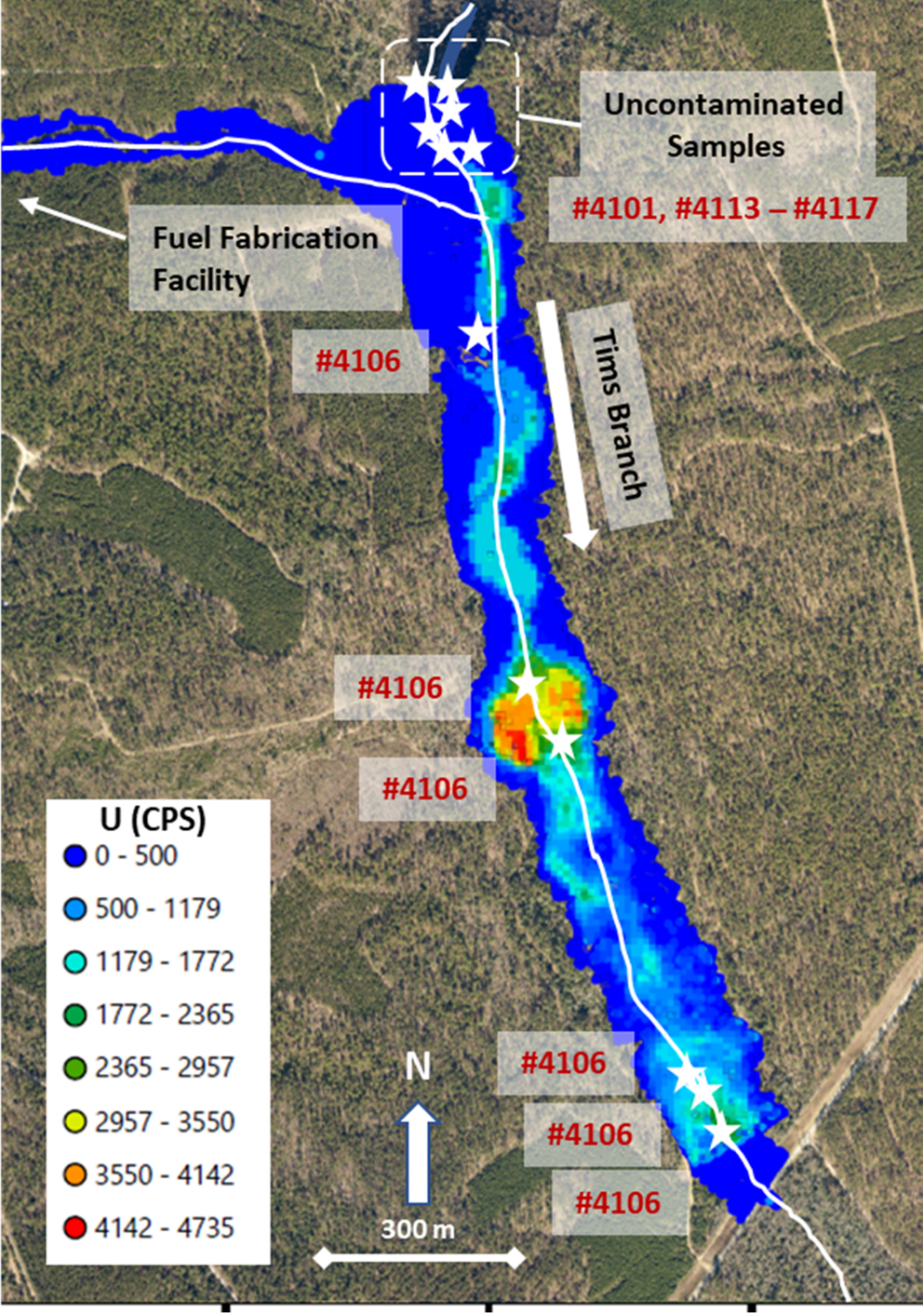
Rhizosphere sample locations (stars and sample IDs) within the uncontaminated (upstream) and contaminated (downstream) portions of the Tims Branch wetland. U map was modified from Kaplan et al.,^37^ where >700,000 γ and X-ray spectra were collected using mobile NaI detectors in backpacks. Reported counts per second (CPS) are from the ^234m^Pa peak (daughter of ^238^U; 766.4 and 1001.0 keV peaks) and were corrected for background levels.

Twelve 1-kg clumps of sediment were collected with a hand spade from a <15 cm depth at U-contaminated and uncontaminated portions of the Tims Branch wetland study site (Figure 1). *Jancus effuses* (Soft Rush) were already growing on the sediments at the time that they were sampled. These plants cannot tolerate long-term saturated moisture conditions and generally grow in areas that are intermittently saturated, which was approximately 0.5–2 m from the stream’s edge. Sampling took place during a summertime dry period and these surface sediments had been unsaturated for months. The samples were placed in zip locked bags and stored in an ice chest until they were transported to a 5 °C refrigerator in the laboratory. Subsequent handling took precautions to maintain the samples under field-moist conditions, but not under an inert atmosphere. As most of the U at our site is found in the surface 5 cm sediment layer,^37^ these results will be applicable to the majority of the U in the study site, as well as at other field sites with surface U contamination. Rhizosphere subsamples were operationally defined as sediments with a distinctive reddish color, originating from the presence of ferric oxyhydroxides and generally within 5 mm of the root (Figure S1). These moist rhizosphere subsamples were manually separated from the root and nonrhizosphere sediment with the use of spatulas, razor blades, and a magnifying desk lamp within 1 week of collecting the samples from the field. Between 0.9 and 1.9 g of field-moist rhizosphere sediments and 10s of grams of nonrhizosphere sediment were collected from a subsample of the 1-kg clump sample. Roots were removed from both fractions. The color-based operational definition was used because it could be unambiguously implemented and perhaps more importantly, it provided well-defined biogeochemical evidence that the roots had chemically impacted the nearby sediments. Furthermore, the Fe redox chemistry in wetlands has a profound effect on the distribution and types of organic matter,^24^ metal chemistry,^38^ and microbial populations.^39^ Other researchers have defined the rhizosphere as the region around roots impacted by changes in gases (e.g., O_2_ or CO_2_), water content, microbial composition, or chemical properties (e.g., organic matter and pH) (reviewed in Waisel^40^), but the disadvantage of these operational definitions is that they are more difficult to implement and they require multiple small-scale measurements.

### General Sediment Characterization

Total C, N, and S were determined by combustion/inferred detection (LECO CNS-2000 analyzer) on air-dried samples. Because these are acidic soils (e.g., Table 1), they contain little inorganic carbon. As such, these measurements can be thought of as measures of organic C, total N, and total S. pH was measured in a 1:1 sediment/water suspension that was permitted to equilibrate for >1 h. Total U, Mn, and Fe sediment concentrations were determined on air-dried samples by digesting 0.25 g of homogenized samples using 90–95 °C heated concentrated HNO_3_ and 30% H_2_O_2_ per the EPA 3050B method.^41^ An aliquot of each digestate was analyzed in a 2% HNO_3_ matrix via inductively coupled plasma-mass spectroscopy (Thermo X-Series II). In addition to a water blank, NIST standard sediment sample #8704 was carried through digestion and ICP-MS analyses. All digestions and analytical analyses were conducted in duplicate.

**Table 1:** Chemical Properties of the Paired Rhizosphere vs Nonrhizosphere Sediment Samples Collected from the Contaminated Sections of the Study Site (Mean ± Standard Deviation)^a^

	nonrhizosphere	rhizosphere
U (mg/kg)	131 ± 155	310 ± 298
pH	4.98 ± 0.31	5.02 ± 0.43
Fe (mg/kg)	8783 ± 5196	21,798 ± 7026^b^
Mn (mg/kg)	72 ± 53	185 ± 186
C (wt %)	2.97 ± 1.24	2.68 ± 1.22
N (wt %)	0.26 ± 0.29	0.23 ± 0.11
S (mg/kg)	356 ± 145	351 ± 81

a Similar data for all 12 paired samples collected from the contaminated and uncontaminated sections of the study site are presented in Table S1.

b Significantly different mean between rhizosphere and nonrhizosphere at *p* ≤ 0.01, degrees of freedom = 5.

Statistics were conducted using the software R.^42^ Paired two-sample *t* tests for means and Pearson correlation coefficients were conducted for either all 12 paired samples or 6 paired contaminated samples when issues were specific to U geochemistry.

### Microbial Community Analysis

16S rRNA sequencing was conducted on six paired samples collected from an uncontaminated portion of the wetland that was immediately upstream from the contamination source (Figure 1) to permit analyses in a laboratory not equipped to handle radiological samples. DNA was extracted from both rhizosphere and nonrhizosphere sediment samples using the MoBio PowerSoil DNA Extraction Kit (Qiagen, Germantown, Maryland). Amplicon libraries were generated from the DNA using Illumina’s 16S Sequencing Library Preparation Protocol. The universal primer pair 515*F*/806R was used for targeting the V4 hypervariable region of bacterial and archaeal 16S rRNA genes.^43−45^ Sequencing was performed by using a 600 cycle MiSeq Reagent Kit v3 (2 × 300 bp) via the Illumina MiSeq platform at the University of Delaware DNA Sequencing and Genotyping Center located at the University of Delaware (Newark, DE).

Sequences were analyzed using the Qiime2 bioinformatics platform (10.1038/s41587-019-0209-9) and processed to remove barcodes, remove poor quality reads, and denoised to remove chimeric sequences using dada2. After processing, the number of sequences per each of the six sets of paired samples for both bacterial and archaeal libraries ranged from 19,475 to 44,170 sequences. Taxonomic assignments were performed using the SILVA 16S rRNA database (Release 138-99). Differences in relative abundances between bacterial and archaeal taxa were assessed at the phylum and genus level.

### X-ray Absorption Spectroscopy

X-ray absorption near-edge structure (XANES) and extended X-ray absorption fine structure (EXAFS) spectra were collected from the sediment fractions at the U L_III_-edge (17,166 eV) in fluorescence mode using a 4-element Vortex detector. The hydrated sediment fractions were packed into drilled plastic slides and sealed with a Kapton film. Samples were transported to the nearby MRCAT/EnviroCAT Insertion Device beamline (Sector 10, Advanced Photon Source)^46^ and measured approximately 2 months after collection from the field under ambient atmosphere and at room temperature. Radiation-induced shifts in the spectra were not detected during measurements made at six fresh locations on the sample. XANES and EXAFS data from U minerals, U complexed in solution, and standards of U(VI) adsorbed to various surfaces such as carboxyl-functionalized beads, iron-oxides, and clays were measured during previous studies at the same beamline.^47−52^ Energy calibration was established by setting the inflection point in the spectrum from a hydrogen uranyl phosphate standard to 17,166 eV and maintained by frequent or concurrent collection of data from the standard.

Fe K-edge (7,112 eV) X-ray absorption spectroscopy, measurements were carried out at the MRCAT/EnviroCAT Bending Magnet beamline (Sector 10, Advanced Photon Source).^53^ The sediment samples from the nonrhizosphere areas of the soil clump were mounted in 1.5-mm-thick plastic slides sealed with Kapton windows and measured at room temperature in fluorescence mode. The samples from the rhizosphere fractions were more concentrated in Fe (Table 1) and were mounted on the Kapton tape and measured at room temperature in transmission mode. Energy calibration was established by setting the inflection point in the spectrum from Fe metal to 7112 eV and maintained by frequent or concurrent collection of data from the standard. Radiation-induced changes in the measurements were monitored by taking 2–3 consecutive spectra at a fresh location. No differences were observed between the spectra, so all scans from each sample were averaged to produce the final spectrum. Analysis of the spectra involved comparisons to Fe standards. The Fe standards, measured at the same beamline in this and previous work,^54−57^ include spectra from goethite, ferrihydrite, hematite, and lepidocrocite that represent Fe in the ferric-oxide fraction. Fe(III) in clay minerals was represented by the following standards: nontronite, hectorite, Illite, and montmorillonite. Fe(III) bound to OM ligands was represented by an Fe(III)-citrate standard. Polycrystalline Fe powders were mounted on the adhesive side of Kapton tape, and their absorption spectra were measured in transmission mode. Clay minerals were homogenized, purified, and size-fractioned using standard procedures and measured as wet pastes mounted in 1.5-mm-thick holders in fluorescence mode. Normalization and background removal of the data was done using the program AUTOBK.^58^ Linear combination fits of the data were performed using the program ATHENA.^59^

### Desorption Distribution Coefficients

Desorption distribution coefficients (*K*_d-desorb_ values) were operationally defined using a sequential extraction protocol^60^ on five whole sediments (i.e., samples that had not been separated into rhizosphere and nonrhizosphere fractions). The whole sediments were used in these measurements because there was not enough mass of the rhizosphere samples to complete the *K*_d-desorb_ measurements. All measurements were conducted in duplicate. A detailed description of the protocol, limitations associated with the protocol, and operational definitions of each of the extractants are provided in the Supporting Information. Briefly, the sequential extraction method included the following: (1) saturated paste extract (*C*_Sat.Paste_; 7-day equilibration in a 1:0.4 DI water:sediment suspension), (2) dilute-acid extract (*C*_Acid_; overnight extraction in a 30:1 liquid/sediment extraction with acetic acid; 0.44 M CH_3_COOH + 0.1 M Ca(NO_3_)_2_), (3) organically bound extract (*C*_Org;_ overnight extraction of sodium pyrophosphate: 0.1 M Na_4_P_2_O_7_ extraction;), (4) amorphous Fe-oxide extract (*C*_AmFeOx_; acidified ammonium oxalate: pH 3 (0.175 M (NH4)_2_C_2_O_4_ + 0.1 M H_2_C_2_O_4_)), and (5) residual (digestion of the remaining sample using aqua regia—1 part HNO_3_:3 parts HCl:1 part H_2_O). Regarding the organically bound extract, Kaplan and Serkiz^60^ demonstrated that sodium pyrophosphate is effective at extracting OM bound to sediment surfaces and does not complex U, presumably due to the molecular structure of pyrophosphate. The *K*_d-desorb_ values (L/kg) were calculated as
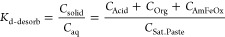
1

The very large 1.0:0.4 DI water:sediment ratio was selected to approximate conditions in a saturated wetland environment. The three extracts used to define *C*_solid_ for *K*_d-desorb_ were intended to represent the U-bound phase that may eventually enter the mobile aqueous phase under a wide range of environmentally relevant conditions. As such, they do not probe U occluded in silicates, aluminosilicates, crystalline Fe-oxides, Mn-oxides, or U that existed as sparingly soluble precipitated phases (i.e., the residual U fraction in the sequential extraction sequence described above). Important advantages of this approach to measuring *K*_d_ values as opposed to the batch-adsorption method, in which the solute is added to an uncontaminated sediment, is that the *K*_d-desorb_ does not require that the researcher guess the solute speciation or concentration, and it has the benefit of using the sediment in which the contaminant was aged under field conditions.

## Results and Discussion

### General Sediment Properties

Among the general soil properties measured, only Fe content was significantly (*p* ≤ 0.01) greater in rhizosphere than in nonrhizosphere fractions (Table 1). Based on earlier hydroponic studies conducted in quarried sand, elevated C, N, and S were anticipated in the rhizosphere.^35^ However, the already very high OM concentrations in these wetland sediments may have masked detection of potentially smaller contributions from the plant and associated microorganisms. While U and Mn contents in the rhizosphere were more than twice those in the nonrhizosphere, they were not statistically different because of the large amount of natural heterogeneity associated with these parameters.

Pooling the data from the rhizosphere and nonrhizosphere samples, Fe and C were significantly (*p* ≤ 0.05) correlated with U concentrations (Table 2; correlation matrices for just the rhizosphere (Table S2) and just the nonrhizosphere (Table S3) fractions are presented in the Supporting Information). This is expected because both sediment components have been shown to strongly bind U.^61^ Significant correlations were also observed between C/N, N/S, and C/S, which may be due to all three constituents existing in these sediments primarily as OM and, as such, are likely covariates.

**Table 2:** Pearson Correlation Coefficients for Sediment Properties Measured in the Rhizosphere and Nonrhizosphere Sediments (Additional Correlation Coefficients for Just the Rhizosphere or the Nonrhizosphere Sediment Fractions Are Presented in Tables S2 and S3)

	U	Fe	Mn	C	N	S
Fe	0.590^a^					
Mn	0.480	0.679^a^				
C	0.813^a^	0.171	0.379			
N	0.519	0.338	0.056	0.645^a^		
S	0.473	0.299	0.076	0.683^a^	0.725^a^	
pH	–0.101	0.419	–0.045	0.045	0.691^a^	0.471

a Significant correlation at *p* ≤ 0.05 with 11 degrees of freedom (critical *r* value ≥0.576).

**Figure 2:**
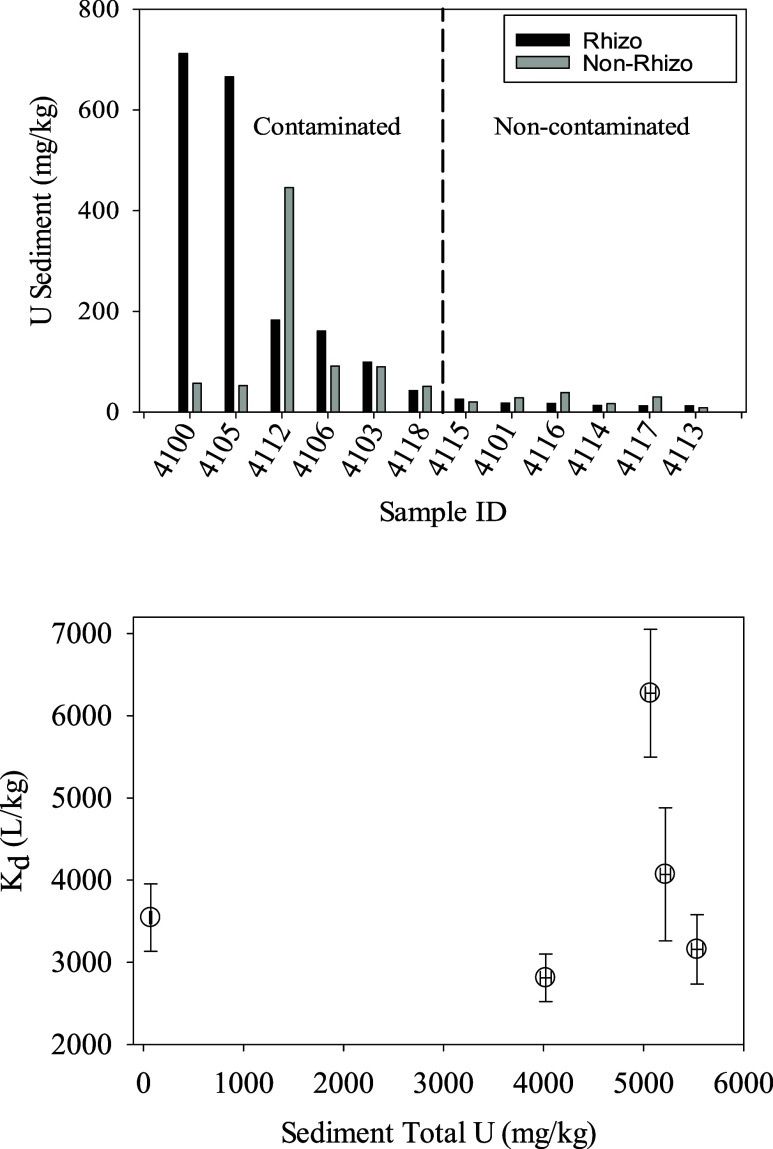
(Top) Uranium in the rhizosphere and nonrhizosphere sediment fraction collected from the contaminated and noncontaminated regions of Tims Branch. (Bottom) Desorption *K*_d_ values (eq 1; units are (mg-U/kg sediment)/(mg-U/L liquid)) as a function of total sediment U concentrations. Error bars represent 1 standard deviation from duplicate measurements conducted on whole sediments (not fractionated). Differences in U concentrations between total and fractionated sediments can be attributed to natural heterogeneity.

While U concentrations in the rhizosphere were on average greater than those in the nonrhizosphere (Table 1), this trend was neither significant (Table 1) nor consistent for all samples (Figure 2). Looking at individual samples (ID – #4100), U concentrations were as much as 12.7 times greater in the rhizosphere (666 mg/kg U) than in the nonrhizosphere (52 mg/kg U). However, there were samples, such as #4112 that had 2.4 times greater U concentrations in the nonrhizosphere (446 mg/kg U) than in the rhizosphere (183 mg/kg U).

### Uranium Desorption Properties

U *K*_d-desorb_ values (eq 1) were estimated from five unfractionated sediment samples (Figure 2). We were unable to conduct desorption tests with the individual sediment fractions because there were insufficient amounts of rhizosphere material. The mean U *K*_d-desorb_ was 3972 ± 1370 L/kg (mg/kg U)/(mg/L U) and had a range from 2812 to 6275 L/kg (Figure 2). These are extremely high *K*_d_ values for U, especially when compared to *K*_d_ values of sediments with carbonate minerals, where U *K*_d_ values are commonly <1 L/kg.^61^ Furthermore, the values did not change as a function of initial total sediment U. These values are consistent with U *K*_d-desorb_ values measured in another U-contaminated wetland on the SRS located along the Savannah River,^60^ where U *K*_d-desorb_ values ranged from 170 to 6493 L/kg, with a mean of 2261 ± 2463 L/kg.

### Microbial Populations

**Figure 3:**
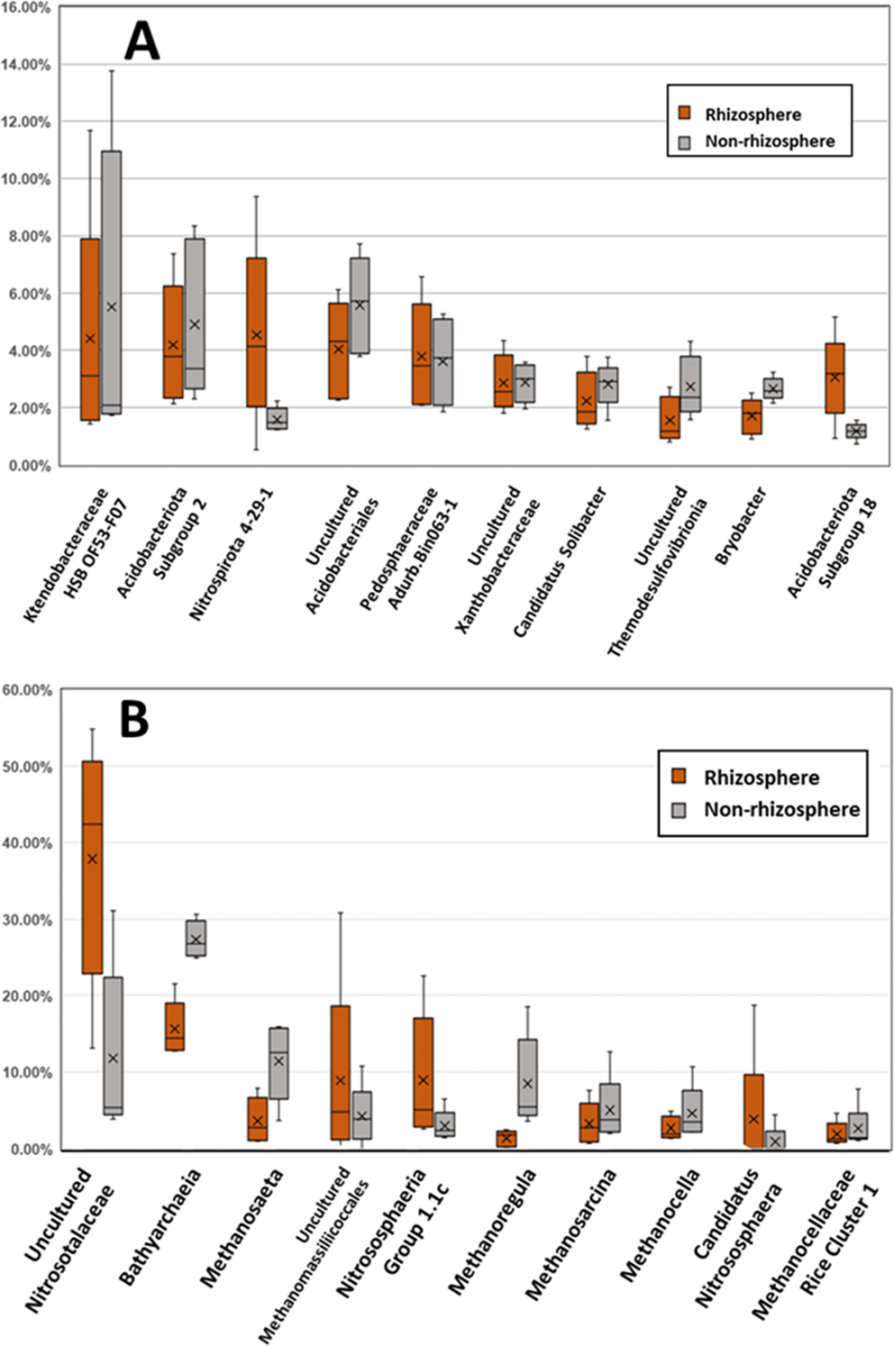
Relative abundance of the most abundant (A) bacterial taxa and (B) archaeal taxa at genus-level assignment from nonrhizosphere (red) and rhizosphere (gray) uncontaminated sediment fractions. Listed are taxonomic assignments of groups classified at the genus level against the SILVA rRNA reference database. Range of five samples indicated by end of whiskers, box plots represent median and quartiles, and “ × ” indicates the mean value.

16S rRNA analyses were conducted to provide additional characterization of the rhizosphere and nonrhizosphere fractions, with the intent that the analyses would provide greater context for explaining U biogeochemistry. Only prokaryotic communities were analyzed and fungi, which can be highly relevant in the rhizosphere and can influence U biogeochemical cycling,^62^ were not analyzed. Greater than 95% of total sequences of taxonomically assigned archaeal 16S rRNA sequences belonged to the phyla *Crenarchaeota*, *Halobacterota*, *Themoplasmatota*, and *Nanoarchaeota* (Figure S2). Higher relative abundances of two methanogenic lineages (genera *Methanosarcina* and *Methanocella*) and the genus *Bathyarchaeia* were observed in nonrhizosphere, but not in the rhizosphere sediments (Figure 3B). Conversely, the relative abundances of sequences related to the genus of *Nitrosotalea* were higher in rhizosphere than the nonrhizosphere samples (Figure 3B). These differences may be associated with higher oxygen concentrations within the rhizosphere region of the wetland. Methanogens are oxygen-sensitive anaerobes, which may explain their lower relative abundances in the rhizosphere.^63,64^*Nitrosotalea* are acidophilic ammonia-oxidizing archaea and may not be negatively affected by the presence of oxygen.^65^

Most archaeal sequences classified at the genus level were *Bathyarchaeia* (Figure 3B), which are associated with detrital degradation of organic material.^66^ The relative abundance of *Bathyarchaeia* was greater in nonrhizosphere samples compared to the rhizosphere. Noteworthy groups within the Archaeal sequences were methanogens (i.e., *Methanosaeta*, *Methanomassiliicoccales*, *Methanoregula*, *Methanosaeta*, *Methanosarcina, Methanocella)*, ammonia-oxidizing archaea (*Nitrososphaeria*, *Nitrosotalea*), and detrital degraders (*Bathyarchaeia)*.

Bacteria phyla detected included the *Acidobacteriota*, *Proteobacteria*, *Chloroflexi*, *Verrucomicrobiota*, *Nitrospirota, Desulfobacterota, Bacteroidota, Planctomycetota, Firmicutes*, *Patescibacteriam Myxococcota*, Candidate phylum Sva0485, and *Actinobacteriota* (Figure S2). The three phyla with the largest relative abundances, *Acidobacteriota*, *Proteobacteria*, and *Chloroflexi*, were also reported in a previous 16S rRNA molecular survey of sediment samples from Tims Branch.^67^ The ten most abundant groups of sequences of bacteria when classified at the genus level across all samples are presented in Figure 3A. Five of these groups are affiliated with the Phylum *Acidobacteriota*, once again emphasizing the potential importance of these organisms in both the rhizosphere and nonrhizosphere. The *Acidobacteriota* possess many relevant traits for the rhizosphere environment, including wide diversity of carbohydrate active enzymes, exopolysaccharide (EPS) production, ability to use various anaerobic electron acceptors (iron, nitrite, nitrate) for anaerobic respiration, ability to act as plant growth-promoting microbes, and variable oxygen sensitivity.^68−70^ Several strains of *Acidobacteriota* have been demonstrated to produce siderophores by chrome azurol S-containing media.^68^ In addition, genomic analyses have identified siderophore-related genes in various members of *Acidobacteriota*.^70^ Most bacterial sequences appear to be heterotrophs, which may contribute to the OM transformation. Organic matter has been identified as a major factor controlling uranium speciation in wetlands,^71,72^ and changes induced by microbial activity on OM composition or moiety distribution may impact U partitioning and speciation.

Previous studies from rhizosphere and nonrhizosphere samples from Tims Branch reported the detection of sulfate-reducing bacteria, Fe(III)-reducing bacteria, and methanogens.^12,36^ Examples of all three groups of organisms were detected in both rhizosphere and nonrhizosphere samples. Iron-reducing bacteria (i.e., *Geobacter* and *Geobacteracaea*), as well as genera of Fe-oxidizing bacteria (*Sideroxydans, Gallionella*, *Leptospirllium*), were detected in both the rhizosphere and nonrhizosphere (Figure S3). Sequences from the genus *Nitrospira* were greater in the rhizosphere, indicating a potential of greater importance for nitrification in the rhizosphere environment. This mirrors the observation of greater relative abundances of ammonia-oxidizing archaea within the rhizosphere (Figure 3B).

Elevated relative abundances of *Nitrosotalea* (Figure 3B), which oxidize ammonia to nitrite using oxygen as terminal electron acceptor and *Nitrospira* (Figure S3), which oxidize nitrite to nitrate, may possibly indicate that nitrification is an important process in the rhizosphere.^65,73^ Previous studies have reported that nitrification and coupled denitrification occur and often at higher rates in the rhizosphere compared to the nonrhizosphere.^74−76^ Sequences related to *Gallionellaceae* were detected in both the rhizosphere and nonrhizosphere (Figure S3). A microbe related to *Gallionellaceae* has been recently reported to couple the oxidation of Fe(II) with nitrate reduction.^77^ Nitrification could increase Fe-oxide formation by providing nitrate, which could be used to oxidize Fe(II) even in the absence of oxygen. Nitrate-linked Fe oxidation may contribute to an extended range of Fe oxidation due to the greater solubility of nitrate compared to oxygen, which is likely more constrained to regions close to the roots.^40^ Microbes related to both Fe oxidation and reduction were detected in both the rhizosphere and nonrhizosphere sediments, but apparently environmental conditions favorable to Fe-oxide formation were only present in the rhizosphere. This could contribute to differences in the binding of radionuclides by oxidized forms of Fe in the rhizosphere environment compared to nonrhizosphere sediments.

### Iron Speciation

**Figure 4:**
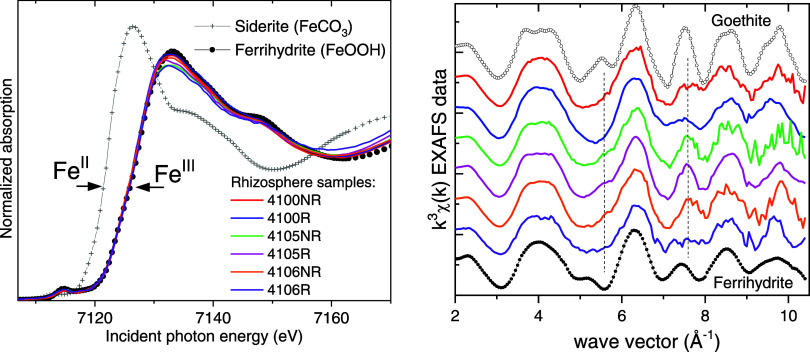
Iron K-edge XANES and EXAFS spectra from paired rhizosphere (R) and nonrhizosphere (NR) samples #4100NR/R, #4105NR/R, and #4106NR/R compared to Fe(II) and Fe(III) standards. Vertical dashed lines in the χ(k) data mark more prominent differences between the R and NR samples that are discussed in the text.

Fe and U XANES and EXAFS spectra were collected from three of the six paired rhizosphere/nonrhizosphere sediment fractions with the greatest U concentrations. The overlay of the Fe XANES edge position and features with the Fe(III) standard indicated that Fe in all samples was in the Fe(III) valence state (Figure 4). This was in part expected because the sediments were collected from the surface unsaturated zone and ambient atmosphere was not excluded during sample collection, preparation, and analysis.

The Fe K-edge EXAFS spectra show differences between the nonrhizosphere and rhizosphere pairs in two of the three analyzed sediments, with the most visible features being around 5.5 and 7.5 Å^–1^ (Figure 4). The spectral differences were quantified using linear combination fits with Fe standards (Figure S4). This approach assumes that the measured spectrum is a weighted average of contributions from several distinct Fe species in the sample and that these species are well-represented by the corresponding standards. All possible combinations with up to four of the nine standards listed in the Methods section were refined against the data (246 fits per data set). Four of the standards (Fh, Gt, SWy-2, and Fe-citrate) were present in the best fits of all data, but in most cases, three components were sufficient to reproduce the data so one of the components minimized to zero (Figure S4).

All samples had Fe assigned to the goethite, Fe(III)-OM, and ferrihydrite fractions, while only sample #4100 had a small Fe fraction in clay structures (spectral weights are shown in Table S4 and Figure S4). On average, ferrihydrite was the dominant form of Fe in the samples, and it tended to exist in greater concentrations in the rhizosphere than in the nonrhizosphere. These conclusions are consistent with previous Mössbauer results of rhizosphere and nonrhizosphere samples collected from the uncontaminated portion of Tims Branch.^12^

### Uranium Speciation

**Figure 5:**
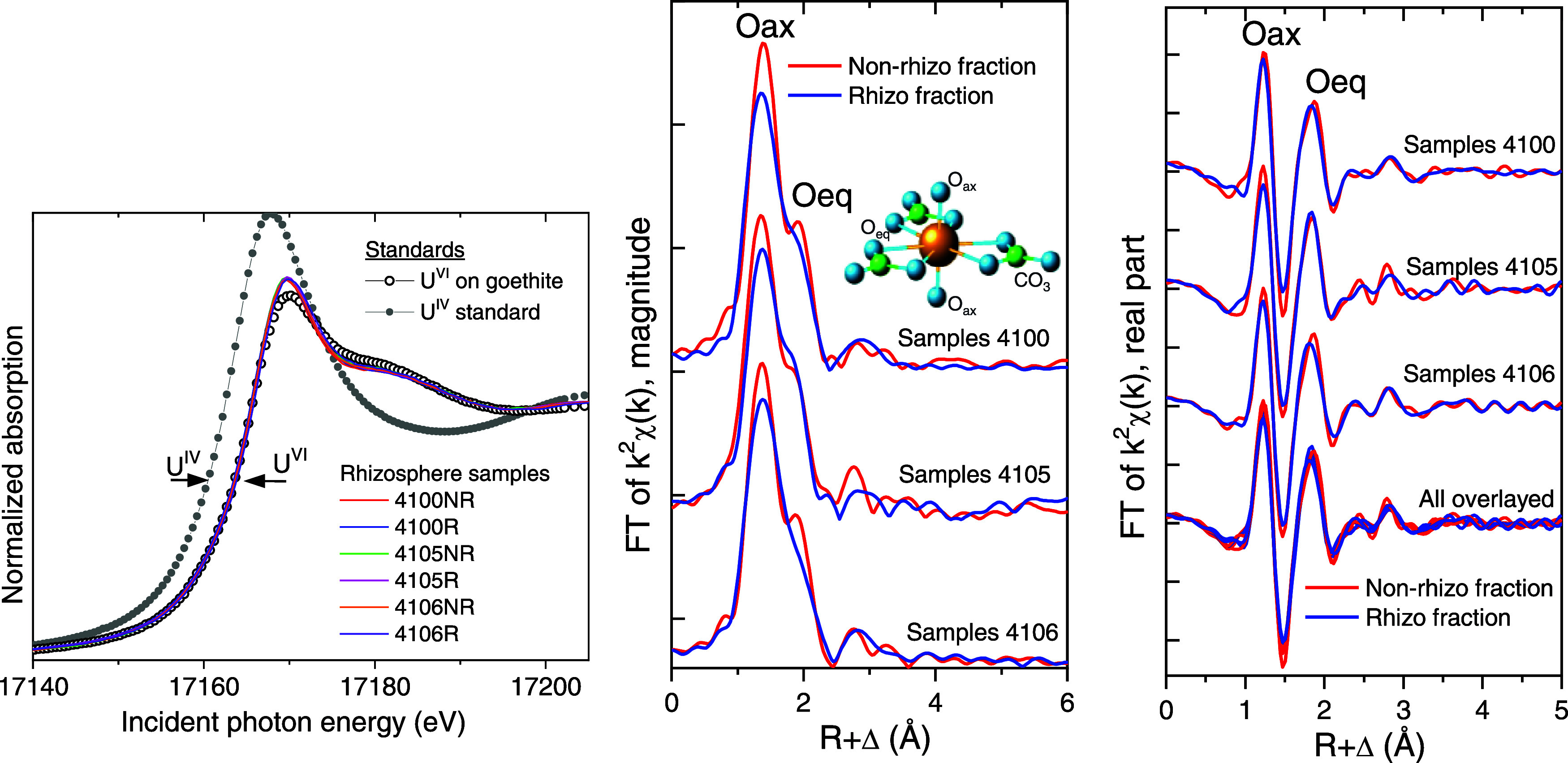
(Left) U L_III_-edge XANES spectra obtained from the nonrhizo (NR) and the rhizo (R) samples compared to U(IV) and U(VI) standards. (Middle and Right) Fourier transforms (FT) of the U L_III_-edge EXAFS spectra obtained from the nonrhizosphere (NR, red) and the rhizosphere (R, blue) samples. The χ(k) data are presented in Figure S6. The inset shows the structure of a uranyl-carbonate complex for illustration and connection to the spectral features.

Figure 5 (and Figure S5) compares the U L_III_-edge XANES and EXAFS spectra obtained from nonrhizosphere (NR, red line) and rhizosphere (R, blue line) fractions. These paired samples were selected because they had the highest U concentrations and would yield the strongest spectral signal (Figure 2). As such, the results may not represent U chemistry in sediments with low U concentrations, especially those sediments where U is primarily of natural origin. The XANES spectra indicate that the U was present as U(VI) in all samples (Figure 5). A consistent trend between the nonrhizosphere and rhizosphere fractions is evident mostly in the real part of the Fourier transform (Figure 5), where each pair of spectra and the overlay of all six spectra show a smaller amplitude of the signal in the axial oxygen (Oax) region and a small contraction of the average equatorial oxygen distance (Oeq) in the rhizosphere fractions. The rest of the smaller-amplitude features (above R + Δ ∼ 2.3 Å) between the paired rhizosphere/nonrhizosphere spectra are similar within the noise of the data.

The U EXAFS data were analyzed by linear combination fits with weighted combinations of spectra from the standards. Because the spectral trends are likely the result of U in two or more sediment compartments contributing in different proportions to the measured spectra, shell-by-shell fits of the paired samples did not yield parameters that would allow identification of the U species (e.g., fits resulted in slightly larger Debye–Waller factor for the Oax shell and a U-Oeq distance contraction of 2.38–2.36 Å for rhizosphere versus nonrhizosphere samples; analysis not shown). A combinatorial fit approach with up to four of the standards listed in the Methods section showed that both the nonrhizosphere and the rhizosphere subsamples are best fit with the three components shown in Figure S7 (i.e., U bound to carboxyl ligands, U bound to DFOB, and U adsorbed on goethite). However, the weights for the U-goethite standard are consistently smaller in the nonrhizosphere than in the rhizosphere subsamples. To determine if this component improves the fit significantly, an additional set of fits without U-goethite is shown in Figure S5. The results are compared in Table S5 and show that the addition of the U-goethite component causes a significant decrease in the R-factor only in the rhizosphere subsamples. Thus, the EXAFS linear combination analyses that about 30–50% of U in the rhizosphere fraction of the sediment is associated with iron-oxides.

The amplitude of the weighed U(VI)–Fe(III)-oxide fit component and the corresponding weight factor were two–three times smaller in the nonrhizosphere versus the rhizosphere samples (Figure S7). The increase in the mineral-adsorbed U(VI) proportion came at the expense of the carboxyl-adsorbed proportion, whereas the siderophore-bound U(VI) component remained relatively constant at 30–50%. This analysis supports the interpretation that there is a significant increase in the proportion of Fe(III)-oxide-associated U(VI) in the rhizosphere relative to the nonrhizosphere, which in turn supports the interpretation that the formation of Fe(III)-oxides near the root zone is responsible for the observed increase in the concentration of U in that region. In keeping with the very minor fit improvement when the U-goethite component was included in the nonrhizosphere linear combination fits, the best averaged description for the U speciation in the nonrhizosphere samples examined here is predominantly OM-coordinated U. In the rhizosphere samples, the U speciation is approximately 60% OM-bound U and 40% mineral-adsorbed U. Note that EXAFS probes only the near-coordination environment of U, so these results do not preclude some OM-U complexes being adsorbed on minerals. For example, Zhang et al.^50^ looked at the interaction between U, the siderophore DFOB, and the Fe-rich nontronite mineral and reported that the DFOB-bound U(VI) was actually sequestered on nontronite clay, presumably by the DFOB bridging between the U and the Fe surface, or by electrostatic/interlayer uptake of the U-DFOB complex in the nontronite. As such, there is little tendency for U bound to solid-phase siderophores to be mobile.

## Implications

U concentrations tended to be greater in the rhizosphere; however, this trend was not consistent. This inconsistency may be due to the observation that U sorbed very strongly to these wetland sediments, with *K*_d-desorb_ values approaching 4000 L/kg on average. This implies that once aqueous U contacted the sediment, there was little likelihood that it would desorb and then resorb in the rhizosphere, which was enriched in reactive goethite and ferrihydrite. Previous hydroponic studies conducted in pure quartz-sand media and in which UO_2_^2+^ was introduced into the aqueous phase, showed as much as a 1500% enrichment of U in the rhizosphere.^35,36^ In these quartz-sand systems, the tendency for the UO_2_^2+^ to bind to the solid phase was appreciably less, possessing a *K*_d_ value in the order of 0.5 L/kg.^78^ By extension, the likelihood that U would desorb from the nonrhizosphere and eventually concentrate in the rhizosphere was much greater in these artificial systems than in the natural wetland system.

The U existed as uranyl UO_2_^2+^ in both sediment fractions. This observation is consistent with the fact that these were unsaturated surface sediment samples. The UO_2_^2+^ was coordinated with Fe(III)-oxides and organic matter. A key difference between the speciation of U in the two sediment fractions was that a larger proportion of the U in the rhizosphere was bound to Fe(III)-oxides than in the nonrhizosphere, which in turn supports the interpretation that the formation of Fe(III)-oxides near the root zone is responsible for the observed increase in the concentration of U in that region. The novelty in this finding is that it was not known whether increased contaminant metal concentrations in the rhizosphere could be attributed to association with the Fe-oxides, OM, or microbes, which are all sediment constituents that are commonly more abundant in the rhizosphere than nonrhizosphere. Together, these results demonstrate that riparian wetlands can strongly bind U, even in the weaker binding U(VI) form, as compared to reduced U(IV). Furthermore, the ability of wetlands to bind U can be enhanced in the rhizosphere, especially in areas where the roots can promote the formation of reactive Fe(III)-oxides.

## Supporting Information Available

Images of rhizosphere, additional data on relative abundances of bacterial and archaeal sequences at phylum level, relative abundance of select bacterial metabolisms; linear combination fits of the Fe K-edge and U L_III_-edge EXAFS spectra obtained from the nonrhizo and rhizo samples; and detailed description of the sequential extraction protocol and results (PDF)
