# Supplementary material for: Uranium Biogeochemistry in the Rhizosphere of a Contaminated Wetland
Source: Environ Sci Technol. 2024 Mar 28;58(14):6381–90. doi: 10.1021/acs.est.3c10481 (PMC11008245; doi:10.1021/acs.est.3c10481)
Supplement: Supplementary file 1 — es3c10481_si_001.pdf [file es3c10481_si_001.pdf]

Supporting Information

Uranium biogeochemistry in the rhizosphere of a contaminated wetland

Daniel I. Kaplan[a*], Maxim I. Boyanov[b,c], Nathaniel A. Losey[d], Peng Lin[a], Chen Xu[e], Edward J. O'Loughlin[b], Peter H. Santschi[e], Wei Xing[a], Wendy W. Kuhne[d], Kenneth M. Kemner[b]

[a] Savannah River Ecology Laboratory, University of Georgia, Aiken, SC 29802, United States
[b] Argonne National Laboratory, Lemont, IL 60439, United States
[c] Chemical Engineering, Bulgarian Academy of Sciences, Sofia 1040, Bulgaria
[d] Savannah River National Laboratory, Aiken, SC 29808, United States
[e] Marine & Coastal Environmental Science, Texas A&M University – Galveston, Galveston, TX 77553, United States

[*] Email: daniel.kaplan@uga.edu

There are 16 pages in the Supporting Information.

List of Figures in Supporting Information

Figure S1. Photographs of field samples showing rhizosphere (reddish areas).
Figure S2. Mean relative abundances of bacterial and archaeal sequences at phylum level. (**A**) Bacterial sequences from non-rhizosphere sediment samples. (**B)** Bacterial sequences from rhizosphere samples. (**C**) Archaeal sequences from non-rhizosphere sediment samples. (**D**) Archaeal sequences from rhizosphere samples.
Figure S3. Relative abundance of select bacterial metabolisms (iron associated (Fe), nitrogen (N) cycling, sulfate reducing (S) and methylotrophic (M)).
Figure S4. Linear combination fits of the Fe K-edge EXAFS data with the 4 components discussed in the text: Fh=ferrihydrite, Gt=goethite, Cit=Fe(III)-citrate, Clay=Fe(III) in SWy-2 clay. Numerical parameters for the fit components are shown next to the scaled and vertically-offset spectra and are also listed in Table S4.
Figure S5. U L_{III}-edge EXAFS spectra obtained from the non-rhizo (NR, red) and the rhizo (R, blue) samples, going from 4100-top to 4106-bottom. (left) $k^2\chi(k)$ data. (right) FT of the $k^2\chi(k)$ data between k = 2.5 and 11 Å^{-1} in the left figure.
Figure S6. Linear combination fits of the U L_{III}-edge data from the non-rhizosphere (NR) and the rhizosphere (R) samples with the 2 components discussed in the text: U-carb=U(VI) adsorbed to carboxyl-functionalized beads and U-DFOB=U(VI) associated with the siderophore DFOB. Numerical parameters for the fit components are shown next to the scaled and vertically-offset spectra and are also listed in Table S5.
Figure S7. Linear combination fits of the U L_{III}-edge data from the non-rhizosphere (NR) and the rhizosphere (R) samples with the 3 components discussed in the text: U-carb=U(VI) adsorbed

to carboxyl-functionalized beads, U-DFOB=U(VI) associated with the siderophore DFOB, U-Gt=U(VI) adsorbed to goethite. Numerical parameters for the fit components are shown next to the scaled and vertically-offset spectra and are also listed in Table S5.

Figure S8. Uranium fractionation (%-wt) of contaminated sediments based on sequential extractions operationally defined in Table S6.

List of Tables in Supporting Information

Table S1. Rhizosphere vs. non-rhizosphere chemical properties from sampled collected in the contaminated and uncontaminated portion of Tims Branch (mean ± standard deviation).

Table S2. Pearson correlation coefficients for sediment properties in only rhizosphere sediments.

Table S3. Pearson correlation coefficients for sediment properties in only non-rhizosphere sediments.

Table S4. Chemical analyses and Fe(III) linear combination fits in three paired non-rhizosphere (NR) and rhizosphere (R) sediment samples. (Uncertainty is ~10 – 15% (±5 – 7%)).

Table S5. Uranyl speciation in the non-rhizosphere (NR) and the rhizosphere (R) samples based on linear combination modeling of U L_{III}-edge EXAFS spectra. Models were either conducted using two components, representing OM-bound U(VI) (a combination of siderophore-bound U(VI) and carboxyl-adsorbed U(VI)), or three components representing OM-bound U(VI) and goethite-adsorbed U(VI). Fit quality illustrated in Figures S6 and S7.

Table S6. Sequential extraction method.

List of Methods in Supporting Information

Uranium Sequential Extraction Method

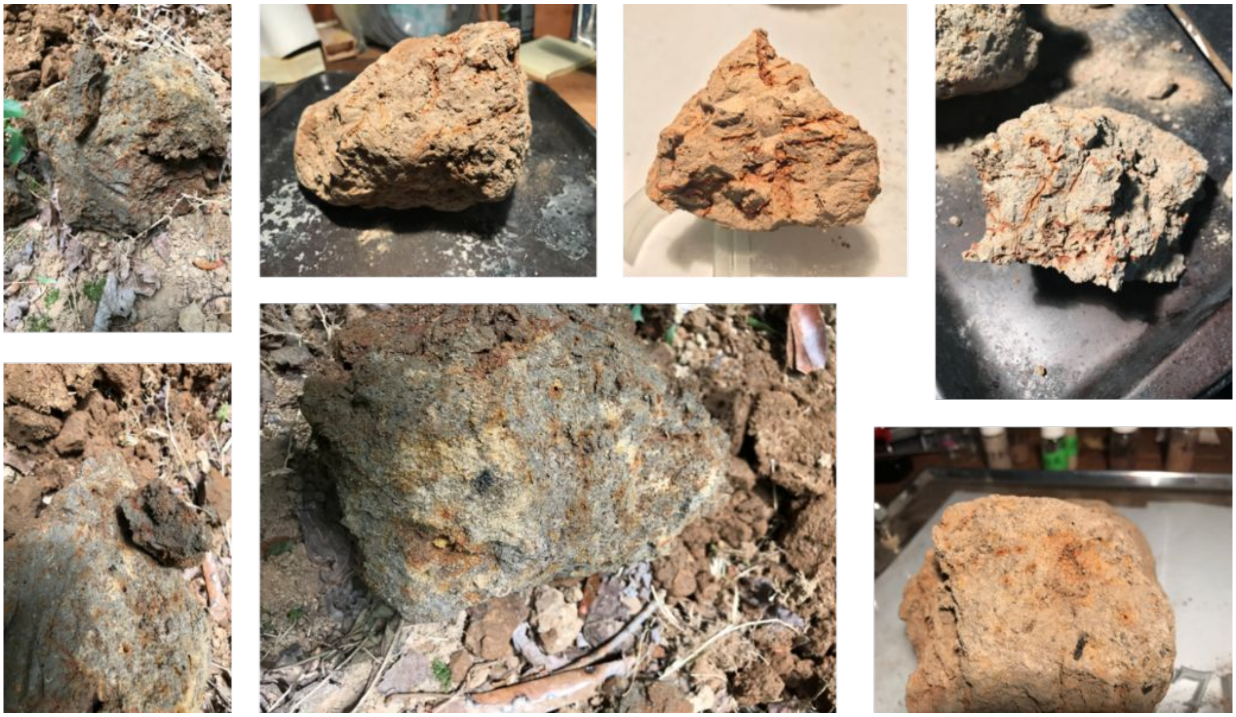

Figure S1. Photographs of field samples showing rhizosphere (reddish areas).

Table S1. Rhizosphere vs. non-rhizosphere chemical properties from sampled collected in the contaminated and uncontaminated portion of Tims Branch (mean ± standard deviation).

	Replicates	Non-rhizosphere	Rhizosphere
U (mg/kg)	12	77 ± 121	163 ± 252
pH	12	4.84 ± 0.10	5.17 ± 0.11
Fe (mg/kg)	12	5274 ± 1480	14466 ± 2641**
Mn (mg/kg)	12	40 ± 14	100 ± 44*
C (wt%)	12	1.80 ± 0.43	1.78 ± 0.40
N (wt%)	12	0.21 ± 0.06	0.23 ± 0.03
S (mg/kg)	12	356 ± 59	351 ± 40

[a] Means and standard deviation for 6 contaminated sediments. U concentrations from sediments from uncontaminated region are not included in these U means (Fig. 3). All other means and standard deviations are from 12 replicates.
**, Significantly different mean between rhizosphere vs. non-rhizosphere at $P < 0.01$, d.f. = 11.
*, Significantly different mean between rhizosphere vs. non-rhizosphere at $P < 0.05$, d.f. = 11.

Table S2. Pearson correlation coefficients for sediment properties in only rhizosphere sediments.

	U	*Fe*	*Mn*	*C*	*N*	*S*
Fe	0.711**					
Mn	0.629*	0.766**				
C	0.854**	0.749**	0.853**			
N	0.113	0.368	0.275	-0.008		
S	0.477	0.912**	0.520	0.698*	0.451	
pH	-0.540	-0.213	-0.305	-0.636*	0.476	0.191

* Significant correlation at $P < 0.05$ with 11 degrees of freedom (critical r value ≥ 0.576).
** Significant correlation at $P < 0.01$ with 11 degrees of freedom (critical r value ≥ 0.708).

Table S3. Pearson correlation coefficients for sediment properties in only non-rhizosphere sediments.

	U	*Fe*	*Mn*	*C*	*N*	*S*
Fe	0.777**					
Mn	0.381	0.802**				
C	0.905**	0.905**	0.580*			
N	0.893**	0.648*	0.153	0.745**		
S	0.678*	0.513	-0.354	0.728**	0.771**	
pH	0.644*	0.616*	0.316	0.666*	0.516	0.751**

* Significant correlation at $P < 0.05$ with 11 degrees of freedom (critical r value ≥ 0.576).
** Significant correlation at $P < 0.01$ with 11 degrees of freedom (critical r value ≥ 0.708).

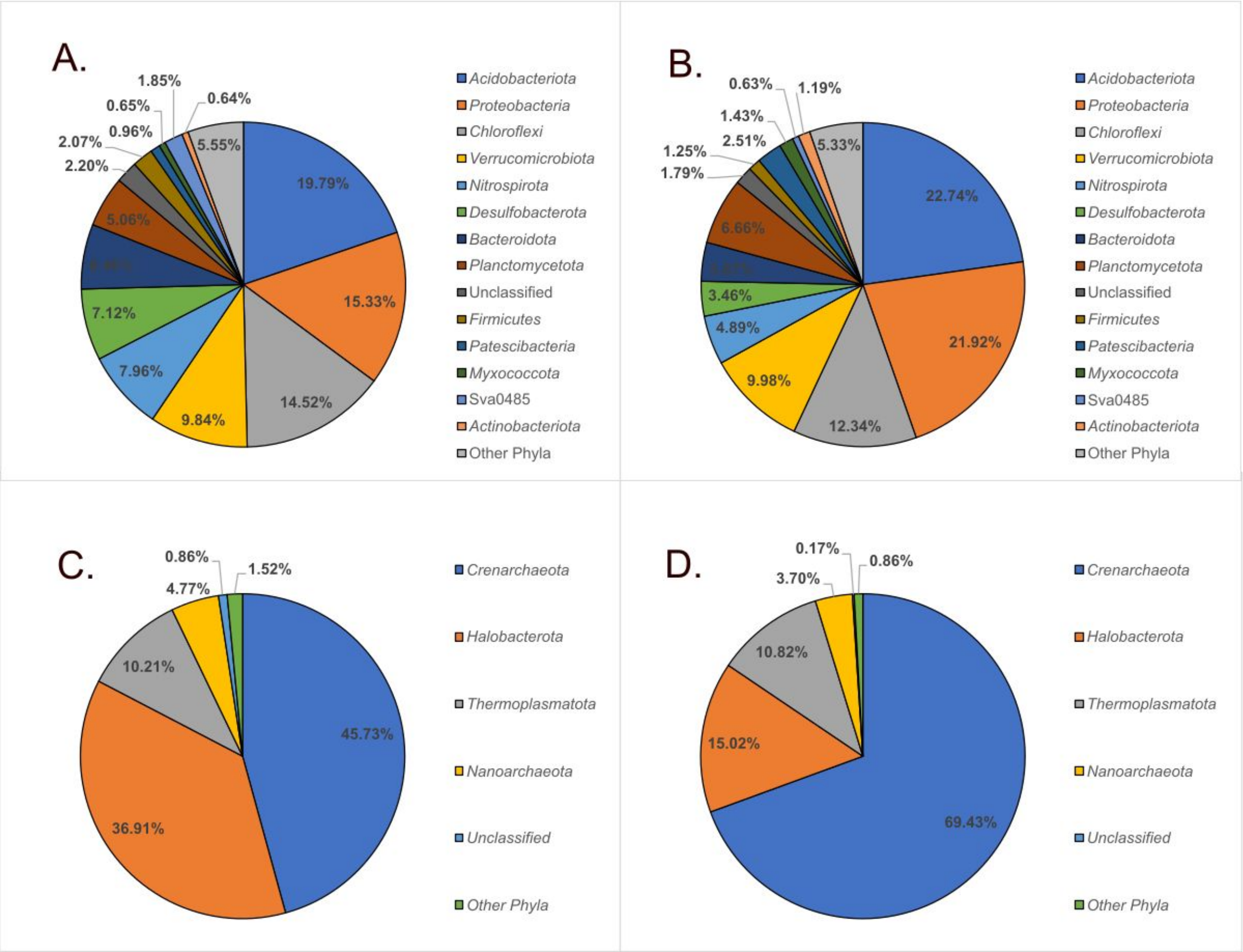

Figure S2. Mean relative abundances of bacterial and archaeal sequences at phylum level. (**A**) Bacterial sequences from non-rhizosphere sediment samples. (**B)** Bacterial sequences from rhizosphere samples. (**C**) Archaeal sequences from non-rhizosphere sediment samples. (**D**) Archaeal sequences from rhizosphere samples.

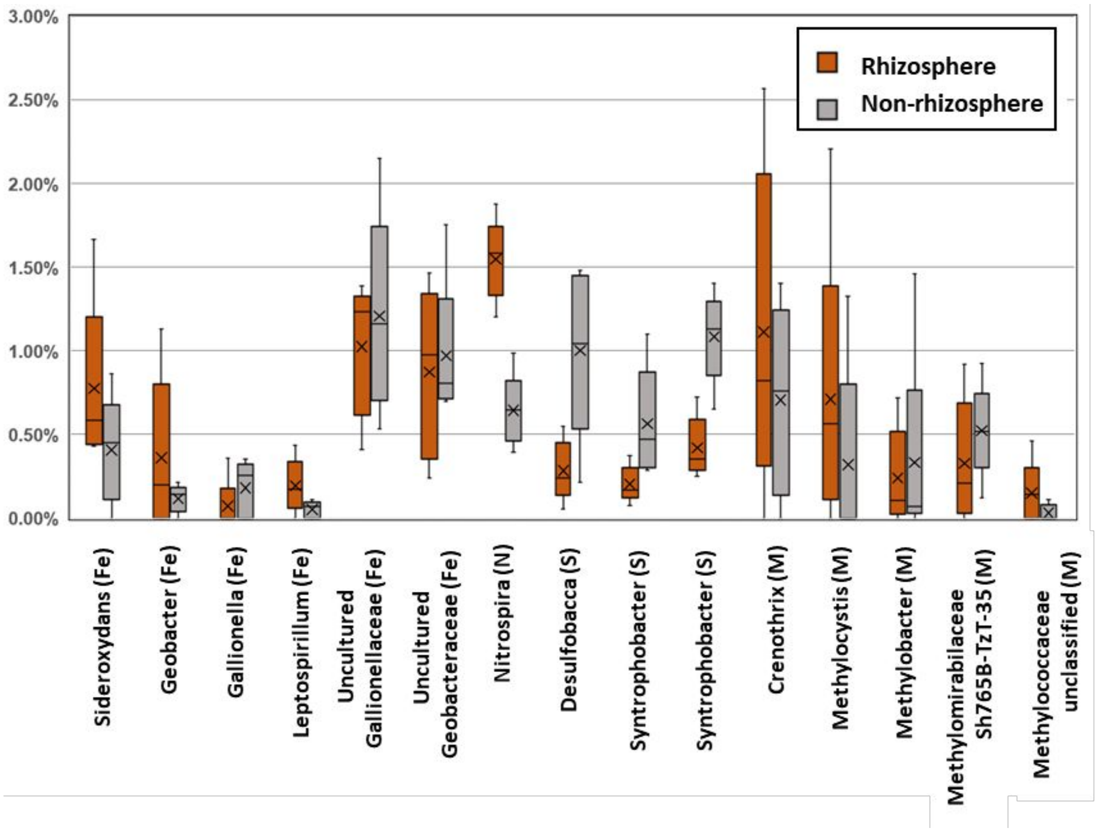

Figure S3. Relative abundance of select bacterial metabolisms (iron associated (Fe), nitrogen (N) cycling, sulfate reducing (S) and methylotrophic (M)). Range of samples indicated by end of whiskers, box plots represent median and quartiles, and '×' indicates mean value.

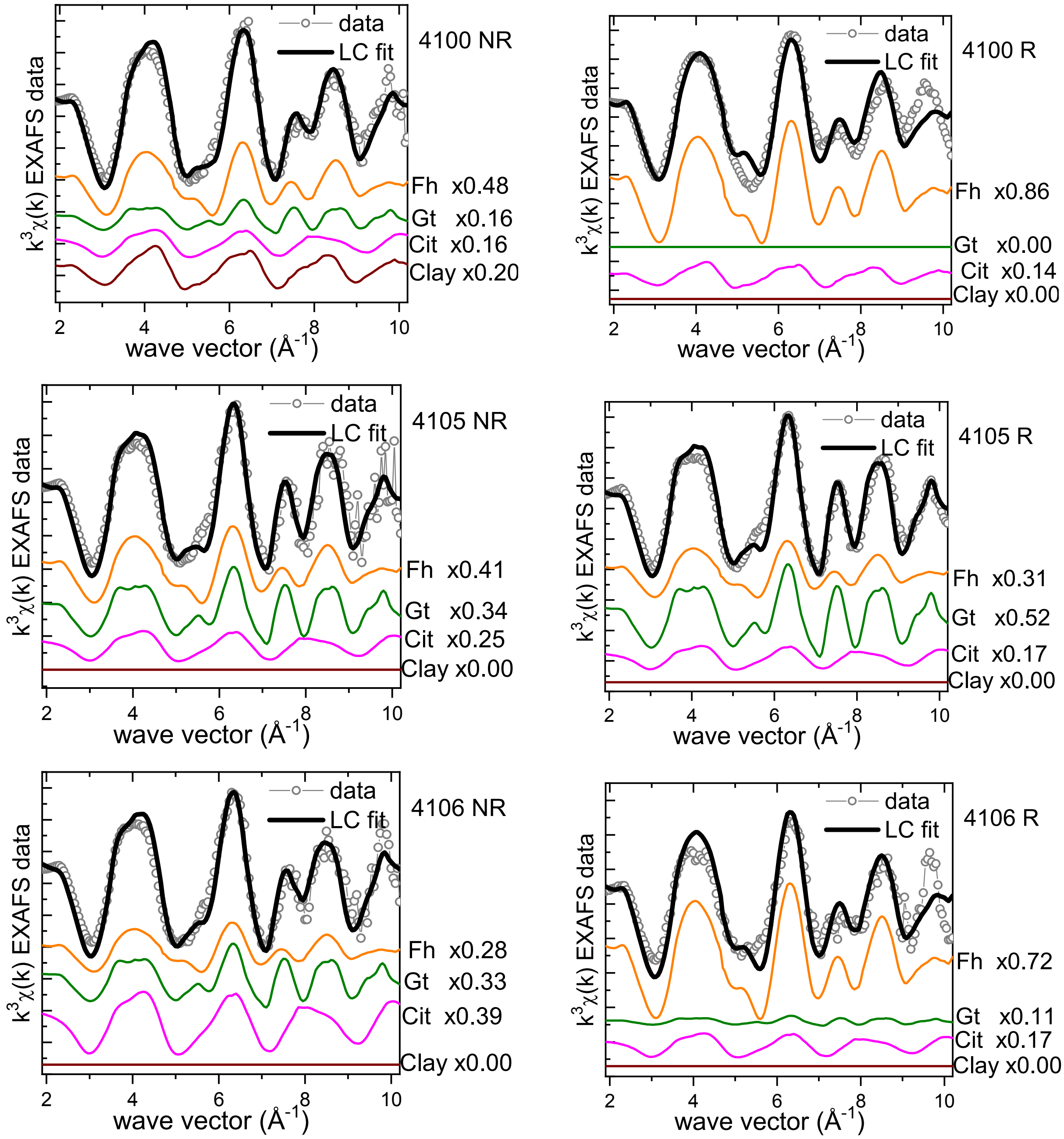

Figure. S3. Linear combination fits of the Fe K-edge EXAFS data with the 4 components discussed in the text: Fh=ferrihydrite, Gt=goethite, Cit=Fe(III)-citrate, Clay=Fe(III) in SWy-2 clay. Numerical parameters for the fit components are shown next to the scaled and vertically-offset spectra and are also listed in Table S4.

Table S4. Chemical analyses and Fe(III) linear combination fits in three paired non-rhizosphere (NR) and rhizosphere (R) sediment samples. (Uncertainty is ~10 – 15% (±5 – 7%)).

				Fe(III) Linear Combination Fit[a]				
Sample ID	Total Fe	Total OC	Total U	Clay Structure	Goethite	Fe(III)-OM	Ferri-hydrite	R-factor[b]
	(mg/kg)	(wt-%)	(mg/kg)	(%)	(%)	(%)	(%)	
4100-NR	2134	NA[c]	56.9	20	16	16	48	0.058
4100-R	15922	NA	712.1	14	0	0	86	0.119
4105-NR	11438	1.97	52.3	0	34	25	41	0.124
4105-R	30057	4.41	665.7	0	52	17	31	0.059
4106-NR	6977	2.55	91.2	0	33	39	28	0.085
4106-R	14883	3.46	160.7	0	11	17	72	0.200
Average NR	6849 ± 4653	2.26 ± 0.41	66.8 ± 21.3	7 ± 12	28 ± 10	27 ± 12	39 ± 10	
Average R	20287± 8477	3.94 ± 0.67	513 ± 306	5 ± 8	21 ± 27	11 ± 10	63 ± 29	

[a] Linear Combination Fit: Reference materials used to estimate the percentages of Fe(III) in the different sediment compartments were a SWy-2 montmorillonite standard for Fe(III) in clay; a Fe(III)-citrate standard for Fe(III)-OM, and a ferrihydrite and goethite standards for Fe(III) in oxides.
[b] R-factor is a measure of the mean square sum of the misfit at each data point. The lower the value, the better the fit.
[c] NA = not available

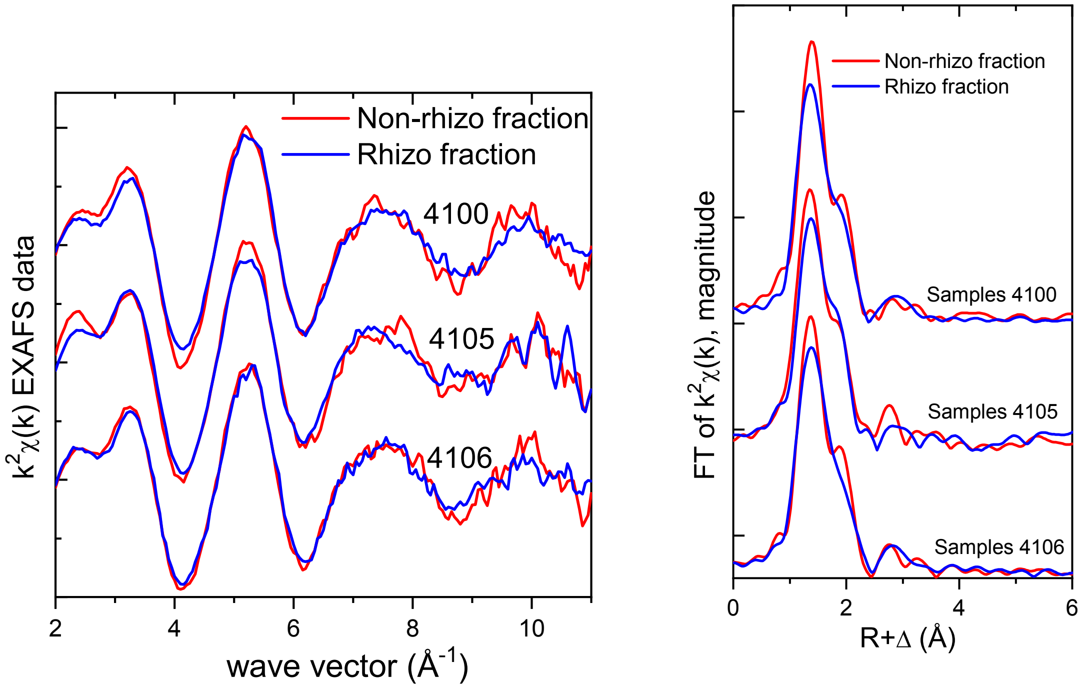

Figure S5. U L_{III}-edge EXAFS spectra obtained from the non-rhizo (NR, red) and the rhizo (R, blue) samples, going from 4100-top to 4106-bottom. (left) $k^2\chi(k)$ data. (right) FT of the $k^2\chi(k)$ data between $k = 2.5$ and 11 Å^{-1} in the left figure. Uncertainties reported by the LC analysis routine were in the range ± 3-7%.

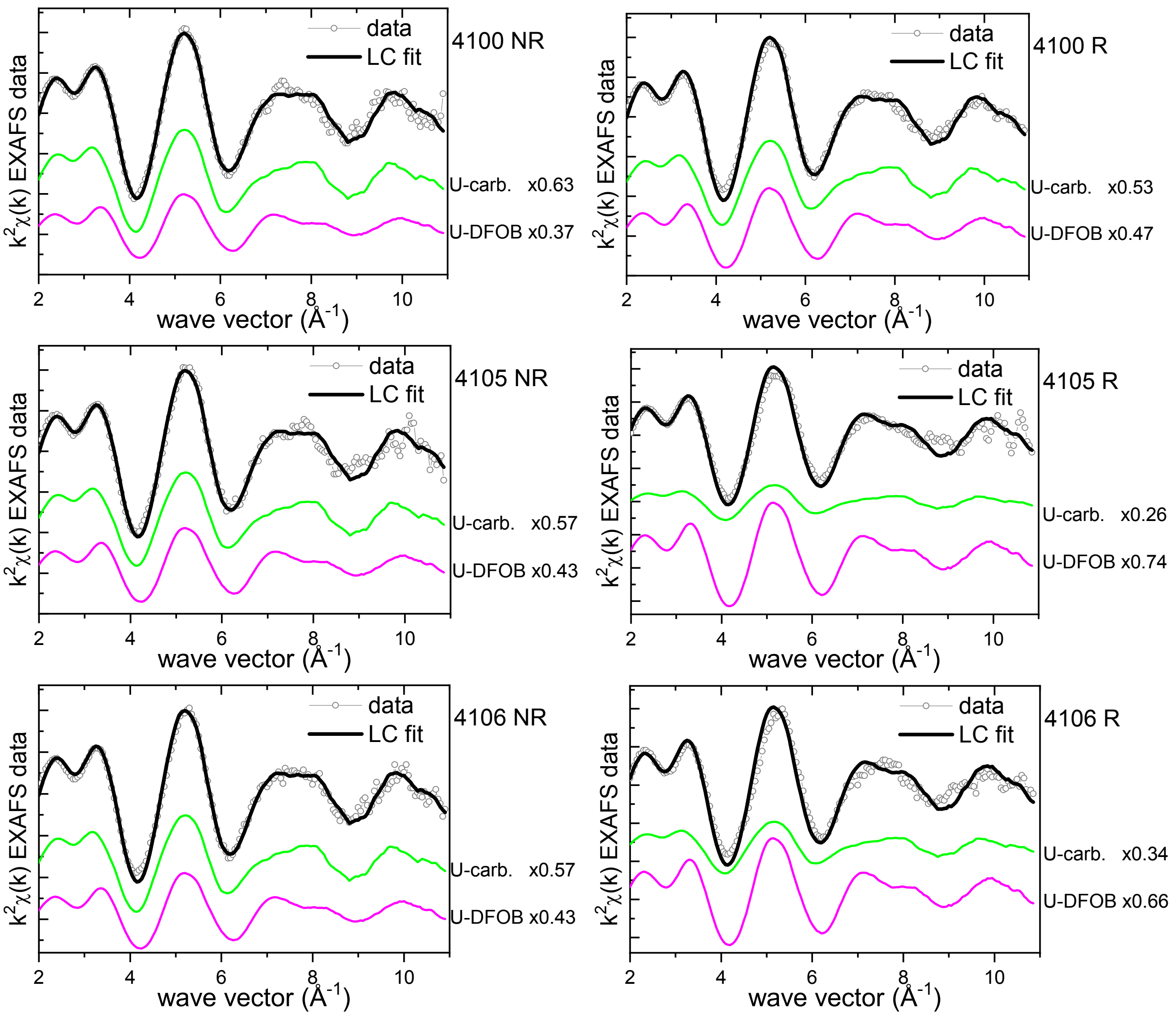

Figure S6. Linear combination fits of the U L_{III}-edge data from the non-rhizosphere (NR) and the rhizosphere (R) samples with the 2 components discussed in the text: U-carb=U(VI) adsorbed to carboxyl-functionalized beads and U-DFOB=U(VI) associated with the siderophore DFOB. Numerical parameters for the fit components are shown next to the scaled and vertically-offset spectra and are also listed in Table S5.

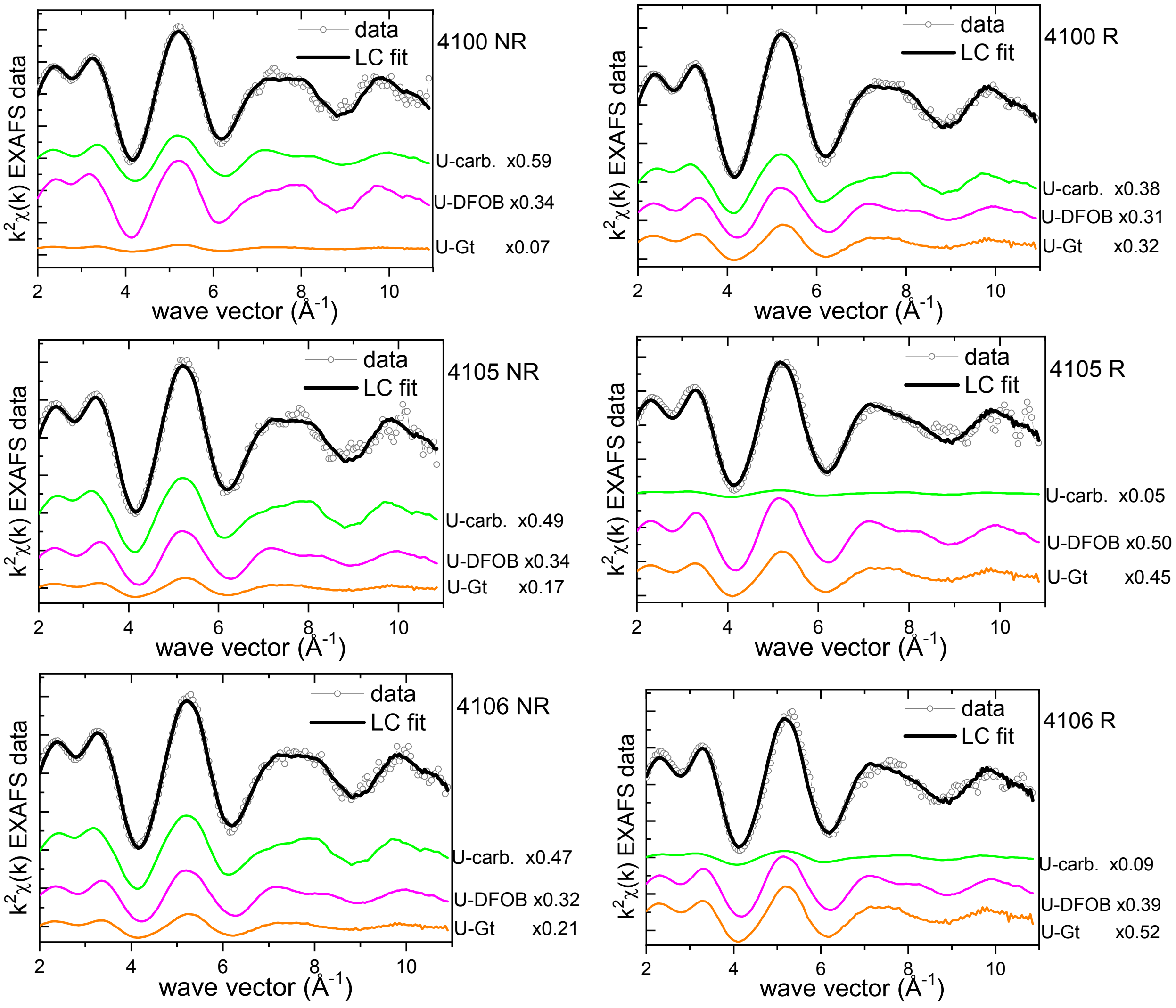

Figure S7. Linear combination fits of the U L_{III}-edge data from the non-rhizosphere (NR) and the rhizosphere (R) samples with the 3 components discussed in the text: U-carb=U(VI) adsorbed to carboxyl-functionalized beads, U-DFOB=U(VI) associated with the siderophore DFOB, U-Gt=U(VI) adsorbed to goethite. Numerical parameters for the fit components are shown next to the scaled and vertically-offset spectra and are also listed in Table S5.

Table S5. Uranyl speciation in the non-rhizosphere (NR) and the rhizosphere (R) samples based on linear combination modeling of U L_{III}-edge EXAFS spectra. Models were either conducted using two components, representing OM-bound U(VI) (a combination of siderophore-bound U(VI) and carboxyl-adsorbed U(VI)), or three components representing OM-bound U(VI) and goethite-adsorbed U(VI). Fit quality illustrated in Figures S6 and S7. Uncertainties reported by the LC analysis routine were in the range ± 3-7%.

Linear Combination Fit ID	Sample	R-factor[a]	Siderophore-bound[b] (%)	Carboxyl-adsorbed[c] (%)	Goethite adsorbed[d] (%)
#1	4100–NR	0.023	37	63	Not included
#2	4100–R	0.025	47	53	Not included
#3	4105–NR	0.038	43	57	Not included
#4	4105–R	0.072	74	26	Not included
#5	4106–NR	0.023	43	57	Not included
#6	4106–R	0.087	66	34	Not included
#7	4100–NR	0.022	34	59	7
#8	4100–R	0.012	31	38	32
#9	4105–NR	0.035	34	49	17
#10	4105–R	0.044	50	5	45
#11	4106–NR	0.018	32	47	21
#12	4106–R	0.050	39	9	52

[a] R-factor is a measure of the mean square sum of the misfit at each data point. The lower the value, the better the fit.

[b] Siderophore bound – Reference spectrum from U(VI) complexed with DFOB. This component represents U(VI) binding to a hydroxamate siderophore or carboxyl organics in the solids.

[c] Carboxyl-adsorbed – Reference spectrum from U(VI) associated with carboxyl-functionalized beads. This component represents U(VI) adsorbed to the carboxyl groups on bacteria or OM.

[d] Goethite-adsorbed – Reference spectrum of U(VI) adsorbed to goethite.

Uranium Sequential Extraction Method

In an attempt to quantify the fraction of contaminant associated with the mineral matrix, some researchers have turned to selective extraction techniques.[1] Importantly, these selective or sequential extraction techniques have experimental limitations.[1, 2] Problems with sequential extractions include incomplete extraction of trace elements or mineral surfaces, non-selectivity of extraction reagents for given soil phases, or re-adsorption of extracted contaminants onto other surfaces. Thus, individual sequential extraction phases may not adequately represent the discrete soil phase to which the contaminant is bound. The actual concentration extracted may only be assumed to be associated with a given operationally defined phase.

The sequential extraction method used in this study was taken from Miller et al.[3] and is a modification of ASTM method D 5074-90[4] and ASTM D-3974-81[5] (Table S6). Five extractions were made from each of the five whole sediments. All tests were conducted in duplicate. These sediments were not separated into rhizosphere and non-rhizosphere because there was insufficient amount of rhizosphere sample to complete the analyses.

Table S6. Sequential extraction method.

Sequence[a]	Extractant: Chemical Composition	Targeted U Fraction	Ref.
1	Saturated past extract: 1:0.4 water:sediment; 7-day equilibration	Aqueous (C_{aq})	6
2	Dilute-acid extract: dilute acetic acid [0.44M CH_3COOH + 0.1 M $Ca(NO_3)_2$]; 0.5g:30mL liquid:sediment; overnight equilibration	Exchangeable (C_{acid})	3
3	Organically bound extract: sodium pyrophosphate [0.1M ($Na_4P_2O_7$]; 0.5g:30mL liquid:sediment	Organic Fraction (C_{Org})	3
4	Amorphous Fe-oxide extract: acidified ammonium oxalate: pH 3 (0.175M $(NH_4)_2C_2O_4$ = 0.1M oxalic acid ($H_2C_2O_4$); 0.5g:30mL liquid:sediment	Amorphous Al- and Fe-oxides (C_{AmFeOx})	3
5	Aqua regia: 1 part HNO_3:3 parts HCl: 1 part H_2O	Structural ($C_{structural}$)	3

[a] Each extract was recovered by centrifugation after the appropriate contact time and then analyzed for ^{238}U by ICP-MS. Between each extraction, the sediment was washed three times for 2 hours with 0.01M $CaCl_2$. After the final wash, the mass of entrained 0.01M $CaCl_2$ solution was gravimetrically determined to account for dilution in the following extraction step.

The first extraction step was the saturated paste. The purpose of this extract was to provide a solution that would approximate sediment pore water. Conducted in duplicate, 0.5 kg of sediment was brought to saturation with D.I. water using the method of Rhoades[6] This method calls for stirring water into the sediments over a course of two to three days, being careful not to leave standing water on the sediment. The saturated paste was allowed to equilibrate for one week. The sediment pore water was then recovered by centrifugation and filtration.

The remaining four extraction steps (Table S6) were conducted using a platform shaker and in duplicate following the procedure of Miller et al.[3] The first three treatments involved selective extractions, whereas the last treatment was total digestion of the remaining sample. The dilute

acid extractant targeted recovery of the exchangeable fraction. The organically bound extractant targeted recovery of U associated with the more readily oxidizable organic matter fraction, leaving organic matter that is harder to oxidize (i.e., organic matter high in lignin content). The amorphous Fe-oxide extractant targeted recovery of U associated with the amorphous Fe-oxides by reductive dissolution of this sediment fraction and leaves intact the more crystalline fraction that requires a stronger reducing agent for dissolution. Finally, after the sediment had undergone the first four extractions, the remaining sediment was totally digested in aqua regia. The U in the aqua regia digest were presumed to be precipitated or in the mineral structure. This fraction is the least likely to contribute to the sorption/desorption process or to enter the mobile aqueous phase. It is important to note that hydrofluoric acid was not used to dissolve the remaining solids, thus the U associated with the alumino-silicates were not included in this fraction. As such, this extraction step may have slightly underestimated the actual remaining U in the residue faction. The selection of this dissolution method was necessary to address safety concerns associated with working with radioactive materials.

A 0.5 g sediment aliquot was treated sequentially with 30 ml of four extractants. Each extract was recovered by centrifugation after the appropriate contact time and then analyzed for ^{238}U by ICP-MS. Between each extraction, the sediment was washed three times for 2 hours with 0.01M $CaCl_2$. After the final wash, the mass of entrained 0.01M $CaCl_2$ solution was gravimetrically determined to account for dilution in the following extraction step.

The U mass extracted in the first three extraction steps were used to calculate the desorption K_d values ($K_{d\text{-}desorb}$) as follows:

$$K_{d-desorb} = \frac{C_{solid}}{C_{aq}} = \frac{C_{Acid} + C_{Org} + C_{AmFeOx}}{C_{Sat.Paste}} \qquad (1)$$

where C_{solid} and C_{aq} are the solid and aqueous concentrations respectively. $A_{acid,}$ C_{Org}, and C_{AmFeOx}, and $C_{Sat.Paste}$ are defined in Table S6 and are the concentration in the acid extractable, organic, amorphic iron oxide and saturated paste extract, respectively. A bar graph of the data is presented in Figure S8.

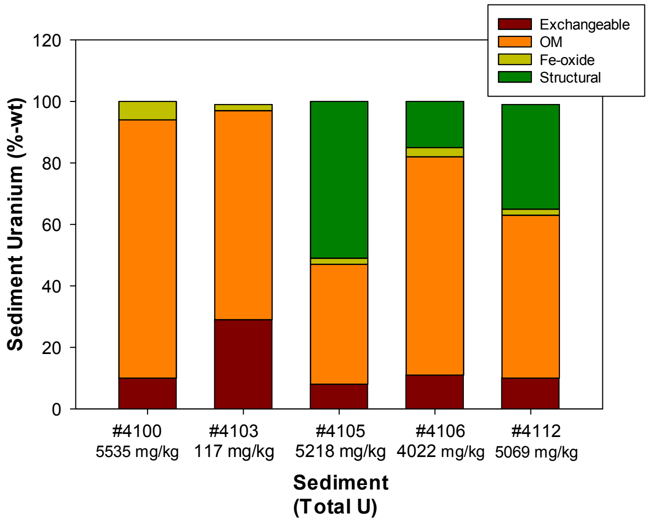

Figure S8. Uranium fractionation (%-wt) of contaminated sediments based on sequential extractions operationally defined in Table S6. The $C_{Sat.Paste}$ for #4100 was 1.75 mg/L, for #4103 was 0.033 mg/L, for #4105 was 0.63 mg/L, for #4106 was 1.22 mg/L, and for #4112 was 0.53 mg/L.

References

1. Tessier, A.; Campbell, P. G. C.; Bisson, M., Sequential Extraction Procedure for the Speciation of Particulate Trace Metals. *Analytical Chemistry* **1979,** *51*, 844-851.
2. Nirel, P. M. V.; Morel, F. M. M., Pitfalls of Sequential Extractions. *Water Resources Research* **1990,** *24*, 1055-1056.
3. Miller, W. P.; Martens, D. C.; Zelazny, L. W.; Kornegay, E. T., Forms of Solid Phase Copper in Copper-enriched Swine Manure. *Journal of Environmental Quality* **1986,** *15*, 69-72.
4. ASTM, Standard Practice for Preparation of Natural-Matrix Sediment Reference Samples for Major and Trace Inorganic Constituents Analysis by Partial Extraction Procedures - D 5074-90. In *The Annual Book of ASTM Standards*, American Society for Testing and Materials: Philadelphia, Pennsylvania, 1990.
5. ASTM, Standard Practices for Extraction of Trace Elements from Sediments - D 3974- 81. In *The Annual Book of ASTM Standards*, American Society for Testing and Materials Philadelphia, Pennsylvania, 1990.
6. Rhoades, J. D., Salinity: Electrical Conductivity and Total Dissolved Solids. In *Methods of Soil Analysis, Part 3, Chemical Methods*, Sparks, D. L., Ed. Soil Science Society Press: Madison, WI, 1996; pp 417-436.
